# Reply to Kuśmierz and Toyoizumi: A network-based explanation of why most COVID-19 infection curves are linear

**DOI:** 10.1073/pnas.2100906118

**Published:** 2021-02-26

**Authors:** Stefan Thurner, Peter Klimek, Rudolf Hanel

**Affiliations:** ^a^Section for Science of Complex Systems, Center for Medical Statistics, Informatics and Intelligent Systems, Medical University of Vienna, A-1090 Vienna, Austria;; ^b^Complexity Science Hub Vienna, A-1080 Vienna, Austria;; ^c^Santa Fe Institute, Santa Fe, NM 85701

In a comment on our recent paper (1), the commentators point out that infection curves on small-world (SW) networks are linear only in the vicinity of the critical point (2) and that infection dynamics on complex or SW networks have already been studied, for instance, in ref. 3. In our work (1), we extend the existing literature by showing, in detail, how an explanation of the apparent sustained algebraic growth regimes observed in many countries during the COVID-19 pandemic involves the structure of the underlying contact network of individuals and how a transition from exponential spreading to a power-law behavior happens when the reproduction number takes values in the vicinity of *R* ≈1.

We are surprised to see that the commentators in ref. 2 try to suggest that, in ref. 1, we demonstrate that the linear behavior should also persist well below the critical reproduction number *R* ≈ 1. We do not do that. Throughout the paper and its extensive supporting information, we emphasize that the derivation of the estimates for the critical connectivity $D_{c}$ (and other arguments) hinges exactly on the assumption that linear behavior can only happen if the reproduction number is close to criticality, that is, *R* ≈ 1, or, equivalently, *D ≈ D*_*c*_.

The critical values *R* ≈ 1 or *D ≈ D*_*c*_ are not sharp, and, as we state explicitly in ref. 1, the approximate formula for $D_{c}$ slightly overestimates the true value. The confusion in ref. 2 might be due to the fact that, at one point (ref. 1, p. 22,687), we state that the critical behavior, that is, linear growth, can be observed when D falls below $D_{c}$. However, we certainly did not indicate with this sentence that we believe that the critical phase extends to values of R far from *R* ≈ 1. Within the context of the entire paper (1) and its supporting information, this is completely clear. It seems that the commentators in ref. 2 took that sentence out of context to create a case that does not exist. For this reason, we strongly object to their statement of having performed a flawed analysis.

In addition, the analysis of close-to-critical spreading on Erdős-Rényi (ER) networks in ref. 2 reveals an important difference between ER and SW networks. A central result in our work (1) is that the critical threshold, above which case numbers grow exponentially in the SW model, is above the levels that are expected from a comparable model with homogeneous mixing of individuals (figure 3*A* in ref. 1). As seen in figure 2*A* in ref. 2, the $D_{c}$ values for ER networks for different settings are clearly below the corresponding $D_{c}$ for SW networks. This difference arises because of network effects that—due to overlapping neighborhoods of nodes—reduce the spread below those levels expected from homogeneous mixing, an effect that also persists, albeit attenuated, in ER networks.

It is fortunate, however, that this comment gives us the opportunity to cite a work that noted the possibility of linear spreading of diseases on SW networks before (figure 5 in ref. 4) and that we were not aware of at the time of composing ref. 1.

## References

[1] S. Thurner, P. Klimek, R. Hanel, A network-based explanation of why most COVID-19 infection curves are linear. Proc. Natl. Acad. Sci. U.S.A. 117, 22684–22689 (2020). 32839315 10.1073/pnas.2010398117 PMC7502715

[2] Ł. Kuśmierz, T. Toyoizumi, Infection curves on small-world networks are linear only in the vicinity of the critical point. Proc. Natl. Acad. Sci. U.S.A., 10.1073/pnas.2024297118 (2021). PMC7958250 33637610

[3] R. M. May, A. L. Lloyd, Infection dynamics on scale-free networks. Phys. Rev. E 64, 066112 (2001). 10.1103/PhysRevE.64.066112 11736241

[4] A. L. Lloyd, S. Valeika, A. Cintrón-Arias, Infection dynamics on small-world networks. Contemp. Math. 410, 209–234 (2006).

